# The Crisis in the Nursing Labour Market: Canadian Policy Perspectives

**DOI:** 10.3390/healthcare11131954

**Published:** 2023-07-06

**Authors:** Andrea Baumann, Mary Crea-Arsenio

**Affiliations:** Global Health, Faculty of Health Sciences, McMaster University, Hamilton, ON L8S 4K1, Canada; mcrea@mcmaster.ca

**Keywords:** Canada, government policy, labour market for care professionals, nurse shortage

## Abstract

The labour market for care professionals has experienced significant changes, resulting in critical shortages globally. Nurses represent the largest share of health workers worldwide; nonetheless, an estimated 13 million more nurses will be needed over the next 10 years. Prior to the pandemic, the domestic supply of nurses in Canada had not kept pace with the ever-increasing demand for services. Pre-pandemic age- and needs-based forecasting models have estimated shortages in an excess of 100,000 nurses nationwide by 2030. While COVID-19 has accelerated the demand for and complexity of service requirements, it has also resulted in losses of healthcare professionals due to an increased sick leave, unprecedented burnout and retirements. This paper examines key factors that have contributed to nursing supply issues in Canada over time and provides examples of policy responses to the present shortage facing the healthcare system. To provide adequate care, the nursing workforce must be stabilized and—more importantly—recognized as critical to the health of the population.

## 1. Introduction

The labour market for care professionals has experienced significant changes, resulting in critical shortages globally. According to the World Health Organization, nurses represent the largest share of health workers, with over 28 million worldwide [1]. A 2022 report by the International Council of Nurses (ICN) estimates that 13 million more nurses will be required over the next decade to fill labour shortages [2]. In Canada, the domestic supply of nurses has not kept pace with the ever-increasing demand for services. Pre-COVID-19 age- and needs-based forecasting models documented a shortage of 60,000 nurses nationwide by 2022 and more than 117,000 by 2030 [3,4]. Although these early models could not predict the impact of a significant health crisis such as COVID-19, they did signal the potential for a future nursing labour shortage. This paper examines key factors that have contributed to nursing supply issues in Canada over time and provides examples of policy responses to the present shortage facing the healthcare system.

The paper begins with a background of the nursing workforce in Canada, followed by a historical review of emerging issues. Using case examples, an analysis of the existing supply is presented. Conclusions are offered with suggestions for future directions.

## 2. Nursing and Canadian Healthcare

National statistics from the Canadian Institute for Health Information (CIHI) indicate that 445,268 nurses were licensed to practice in Canada in 2021 [5]. Nurses work in several settings, including acute care hospitals, long-term care facilities and community organizations. However, the largest employer is the hospital sector. Nearly two-thirds of hospital expenses are related to employee compensation, with nursing making up the greatest share [6]. There are differences across the country in terms of nursing roles, contracts and working conditions. For example, nurses working in specialty areas such as critical care are responsible for the direct patient care of highly complex patients, while nurses working long-term care are responsible for oversight. Furthermore, full-time employment varies across the provinces and territories, with Ontario and Québec having the highest percentage of nurses employed in a full-time capacity [5].

Nursing is one of 30 regulated health professions in Canada under provincial/territorial jurisdiction. Nursing regulators are responsible for overseeing and ensuring the safe practice of nursing in the public interest. Their role involves setting entry to practice requirements, ensuring registration requirements are met, maintaining a database of nurses eligible to practice, monitoring continuing competence and enforcing standards of practice [7]. Regulatory practices vary across the country and can have a direct impact on nursing supply. As Leslie et al. note, “serving the public interest through these regulatory functions exposes potentially conflicting policy tensions, such as public versus professional interests, transparency versus privacy, and accountability versus flexibility” [8] (p. 30). The pandemic exposed system vulnerabilities and highlighted the need for urgent change. Regulators across the country have created initiatives to augment the existing nurse supply.

## 3. Historical Nursing Supply Issues

Several factors have contributed to the shifting trends in nurse supply over the past three decades. These include healthcare restructuring, economic recessions and unanticipated events such as the SARS outbreak in 2003 and the COVID-19 pandemic. The latter two intensified supply issues within the labour market for care professionals. Historically, labour shortages were seen as cyclical and were met with a variety of policy responses. The traditional focus was on the integration of new nurses and retention initiatives.

For example, in the late 1990s, an economic recession led to healthcare restructuring and a loss of nurses from the Canadian labour market [9]. At the time, fiscal pressures resulted in a reduction in full-time nurse positions, nurses being hired into part-time and casual contracts and opting for employment in the United States. In the province of Ontario, a nursing task force was established to examine the consequences of healthcare reform on the delivery of nursing services and the profession overall [9,10]. The task force stressed the need to stabilize the provincial nursing workforce and recommended the creation of new nursing positions and increasing full-time employment.

The SARS outbreak caused substantial concern for policymakers and emphasized the lack of surge capacity in the healthcare system [11]. The casualization of the nursing workforce and an increased use of agency staff, which were status quo, resulted in care issues. Due to the already reduced number of full-time nursing positions, many nurses were working in part-time positions across hospital sites. The National Advisory Committee on SARS and Public Health focused on “the importance of a stable and permanent workforce, rather than reliance on more costly agency personnel” [12] (p. 153). This policy recommendation was reinforced by the Ontario Expert Panel on SARS and Infectious Disease Control, which strongly advised that the government focus on “establish[ing] sustainable employment strategies for nurses and other healthcare workers to increase the availability of full-time employment” [13] (p. 189).

From 2003 to 2006, the province of Ontario invested in the nursing workforce by creating new full-time positions, increasing the number of nurses hired full time in hospitals and long-term care facilities and converting part-time and casual positions into full-time employment [14]. The 2008 financial crisis led to widespread layoffs in various sectors, including manufacturing, finance, agriculture and trade, but not healthcare [15]. As a result, many nurses became primary wage earners. This facilitated the movement of the policy direction in some provinces toward a better ratio of full-time to part-time employment within the nursing workforce.

In the ensuing years, the policy focus shifted to recruitment and retention. Evidence regarding the aging population and the increased morbidity of the population, combined with the outcomes of forecasting models, indicated that Canada would once again enter an acute shortage of nurses. COVID-19 exacerbated this shortage and accelerated the demand for and complexity of service requirements. As the pandemic has subsided, governments have begun to look at the health workforce sequelae. An increased sick leave, unprecedented burnout and early retirements have resulted in losses of health human resources. This has heightened government recognition that the consistent versus episodic tracking of workforce data is paramount for a comprehensive understanding of the depth of this issue, which has persisted over decades.

## 4. The Nursing Workforce and the Importance of Data

To practise as a regulated nurse in Canada, an annual registration with the provincial/territorial regulatory body is mandatory. Nurses complete a registration form that includes demographic, geographic education and current employment information [16]. Each year, the provincial/territorial regulatory bodies submit a set of standardized data to the CIHI. The data elements are standardized to allow for comparisons between international and national jurisdictions. An examination of the nursing workforce data provides insight into how a large cohort is affected by external factors such as crises, workforce-planning policies, shifts in educational requirements and changes in the economy.

A snapshot of Ontario demonstrates that tracking annual information can reveal trends that require policy action. Prior to COVID-19, there was a convergence of evidence reflecting an imbalance between the supply of nurses and demand for their services [11]. The domestic supply of nurses is primarily produced by universities and community colleges, but is augmented by internationally educated nurses (IENs), who account for 13% of the overall supply [17].

The international supply consists of IENs who migrate to Canada as permanent residents [18]. Already certified to practice in their country of origin, they enter as economic or skilled workers, as family class members and, occasionally, as refugees. Although they are an important health human resource, they are not eligible to practice on arrival [19]. They must first undergo an assessment of their foreign credentials to support registration/licensure by the provincial/territorial nursing regulatory bodies.

**Figure 1:**
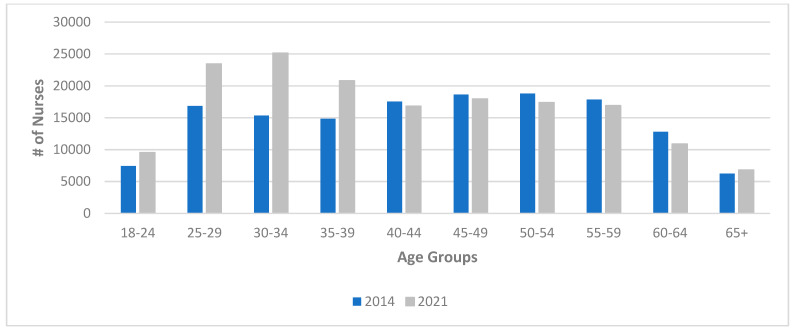
Age distribution of employed nurses in Ontario, 2014 and 2021. Source: College of Nurses of Ontario (2022). Registration statistics report [18].

Tracking annual information also demonstrates changes in nursing demographics. The College of Nurses of Ontario (CNO) is the “governing body for Registered Nurses (RNs), Registered Practical Nurses (RPNs) and Nurse Practitioners (NPs) in Ontario,” and registration with the CNO is a requirement for practice within the province [7]. As shown in Figure 1, membership in the CNO grew between 2014 and 2021. However, there was a significant demographic shift, with younger nurses becoming the largest cohort in the domestic nursing supply.

Concomitantly, the data indicate that the number of non-practising members increased by 60% from 5364 in 2014 to 13,500 in 2019 [20]. It was evident that a serious nursing supply issue was emerging prior to the pandemic. During the pandemic, the labour market was severely affected by the requirement to redeploy staff across all healthcare sectors. Evidence confirmed that nurses were taken from acute care and placed into long-term care, which has had persistent issues with short staffing [21,22]. The demand on the nursing supply continued with reassignments such as work in vaccination clinics and redeployment from intensive care units to operating rooms. As the strain on the existing supply continued, evidence of increased sick time and resignations emerged.

Prior to COVID-19, a research group working with 1200 healthcare employers in Ontario noted that they were experiencing staffing shortages and recruitment difficulties [9]. A longitudinal survey of nursing job postings in Ontario revealed a significant increase in the number of vacancies between 2019 and 2021. As shown in Table 1, there was a 100% increase in the number of job postings during this time. Even then, the domestic supply was insufficient to fill the almost 154,400 RN and 27,800 licensed practical nurse (LPN) job openings projected across Canada [23,24].

When tracking supply, the absolute number of nurses and the number of those active in the labour market are examined, but tracking true vacancies in hospitals has been difficult because of cost-containment efforts and internal turnover. Artificial intelligence (AI) advances have made it easier to calculate real-time vacancy data. Using advanced algorithms, popular job posting websites are scraped daily. This type of information provides an additional crude estimate of the number of nurses required in the health system at any given time.

## 5. The Movement to International Recruitment

Central to the government’s post-pandemic economic recovery policy is an increase in immigration levels [25]. Thus, it is not surprising that provincial policies have emphasized the importance of IENs. As early as 2007, Baumann and colleagues began investigating the advantages of hiring highly skilled, experienced IENs and the considerable difficulties they had obtaining commensurate employment [19,26,27]. International labour organizations have expressed concern about countries actively recruiting nurses, thus depleting the local supply. The ICN (2003), for example, is encouraging “high-income countries … to be self-sufficient in creating enough nurses to meet their populations’ needs” [28] (§2). Canada respected this policy direction until healthcare organizations were faced with an unprecedented shortage of nurses. Historically, most IENs entered the country as secondary migrants under the family class immigration stream. However, due to the critical labour shortages, some smaller provinces have begun actively recruiting nurses in the overseas market [29]. Despite the ICN’s call for an increase in domestic supply, Ontario, which is Canada’s most populous province, had only an 8% increase in the number of new graduates between 2021 and 2022 [20]. The growth has primarily been in the international category, with a 40% increase in the number of IENs over the same period [20].

## 6. Task Shifting—The Rise of the Alternative Worker

Another policy response to nursing shortages has been to increase the number of unregulated workers. This trend is seen by some as not an augmentation but rather a replacement for certain aspects of nursing work. This cadre of workers have physical and environmental responsibilities such as cleaning equipment and making beds. They also focus on meeting basic care needs such as bathing and feeding patients. During the pandemic, initiatives were developed to enhance the supply of personal support workers (PSWs) and healthcare aides. For example, the government of Alberta created tuition and workplace bursary programs for healthcare aides [28]. Ontario introduced the PSW Return of Service Program to recruit 1000 new PSWs by offering a CAD 5000 incentive contingent upon a commitment to work in a long-term care home for at least six months [30]. Two other provinces offered over a 19% wage increase and guaranteed employment in the long-term care sector to anyone interested in completing the PSW training program [31]. A concern with this type of policy response is that it leads to the diminution of professional care in the healthcare environment. By creating initiatives to increase the supply of unregulated healthcare workers, the number of professional nurses involved in direct care will begin to diminish.

## 7. The Required Nurse Supply: Where Are We Now?

The supply of nurses must include a balance of domestic production and IENs. Not only has the domestic supply not kept pace with the growing demand for nurses, but there has also been a lag in the integration of new graduates into the healthcare system. Workforce integration is defined as “the process by which nurses enter the workforce efficiently, effectively and with productive employment” [10] (p. 49). This means employing nurses in a timely fashion and offering them targeted incentives that encourage their retention. For employment to be productive, it must address nurses’ preferences for full-time employment and provide career supports. Additional research has identified full-time employment and extended orientation as two key factors that affect new nurse integration [9,32,33].

**Figure 2:**
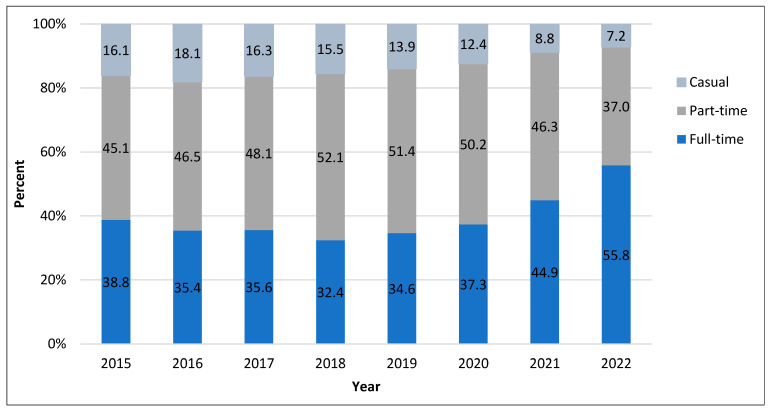
Work status of new nurses in Ontario, 2015–2022. Source: College of Nurses of Ontario (2022). Registration statistics report [18].

Figure 2 shows the percentage of new nurses by work status in Ontario. In the years leading up to the pandemic, only 32% of new graduates were employed in full-time positions [18].

During an economic downturn, new graduate nurses are among the first in the labour force to face layoffs. The availability of positions for new graduates may also decrease due to an increased supply of nurses but a reduced demand for their services. In Ontario, government initiatives have provided incentives to healthcare employers to accelerate the hiring and integration of new graduates. An example of an early policy initiative is the Nursing Graduate Guarantee, the goal of which is to incentivize employers to hire new graduates into their workforces and provide them with an extended orientation and mentorship. Evidence demonstrates that this type of initiative helps new nurses transition to the workforce and remain in the workforce [34].

## 8. The Pandemic Effect—Policy Perspectives

Several targeted government initiatives emerged during the pandemic to address the acute staffing crisis. These initiatives focus on increasing recruitment and improving retention of the health workforce across the acute care and long-term care sectors. Initiatives directed at student nurses include increasing educational seats and funding programs that allow student nurses to work under the mentorship of regulated nursing staff. For example, in British Columbia, over 602 new nursing seats were added to public post-secondary institutions [34]. In Nova Scotia, more than 200 seats have been added to nursing programs [35].

Alberta, British Columbia and Québec have implemented student nurse programs to allow student nurses to work in healthcare [36,37,38]. In addition, Québec has opened its exam for graduates from New Brunswick to become registered in Québec [39], while British Columbia has rolled out new graduate nurse initiatives for certain nursing specialties and at certain regional health authorities [40,41,42].

In Ontario, two programs for nursing students have received government funding. The first is the Extern Program, which “reimburses selected Ontario hospitals for the cost of employing externs and extern mentor/coordinators (EMCs) to address COVID-19-related health human resources challenges” [43] (p. 3). The second is the Preceptor Resource and Education Program for Long-Term Care (PREP LTC), which provides clinical placements for nursing students as part of the government’s plan to recruit and retain long-term care staff [44].

Initiatives also focus on the recruitment and retention of new graduate nurses. In Ontario, the Tuition Support Program for Nurses (TSPN) provides tuition reimbursement for nursing education to recent nursing graduates from rural and remote communities to increase recruitment in these underserved areas [45]. Furthermore, many provinces are providing incentive bonuses ranging from CAD 5000 to 25,000 for a specified term of service [46,47,48,49]. These bonuses were not available to nurses prior to the pandemic.

## 9. Conclusions

A historical review of the Canadian nursing workforce indicates that demand and supply issues require constant monitoring. While governments do provide policy responses in times of crisis, they need to be sustained as the workforce stabilizes. This article demonstrates that in Canada, the indicators of a shortage occur long before a crisis emerges. The COVID-19 pandemic exacerbated existing shortages and it became evident that the domestic nursing supply was significantly lacking. The government’s response was to increase the number of IENs to offset the shortage. Policies were implemented to accelerate their registration and integration into employment. Furthermore, provinces began selected overseas recruitment efforts. However, this is not enough. Investment in the domestic supply needs to be a focus of educational policy. Research highlights the importance of continual “financial, political, public and professional support” for nurses [9] (p. 8) and the need to include them in health policy formation [50,51,52]. To provide adequate care, the nursing workforce must be stabilized and—more importantly—recognized as critical to the health of the population.

A future direction for the government is the need to focus on a balance between the domestic and international supply of nurses. Within Canada, there have been marginal increases in the nursing educational sector to meet the growing demand over time. The lack of planning on the domestic side has resulted in a shortfall of nurses. Governments need to increase nursing educational seats across the country with a concomitant focus on increasing the supply of nursing educators and leaders.

## Figures and Tables

**Table 1:** Ontario job postings by nurse category, 2019–2021.

Nurse Category	2019	2020	2021
Registered Nurse	3479	5131	6647
Registered Nurse/Registered Practical Nurse	88	416	415
Registered Practical Nurse	1951	3162	3912
Total	5518	8709	10,974

Source: Database of job postings for RNs and RPNs in Ontario from 1 September to 30 November 2019, 2020 and 2021 (Unpublished data).

## Data Availability

Not applicable.
